# Impact of MEK inhibition on T-cell infiltration and function after radiotherapy in KRAS-mutant lung cancer

**DOI:** 10.3389/fimmu.2025.1663502

**Published:** 2025-11-24

**Authors:** Yawen Zheng, Chen Su, Jiachao Pan, Yufeng Wang, Mengmeng Zhao, Mingyan Zhang, Peng Yan, Ning Liu, Meili Sun

**Affiliations:** 1 Department of Oncology, Central Hospital Affiliated to Shandong First Medical University, Jinan, Shandong, China; 2 Research Center for Translational Medicine, Central Hospital Affiliated to Shandong First Medical University, Jinan, Shandong, China; 3 Department of Gastroenterology, Central Hospital Affiliated to Shandong First Medical University, Jinan, Shandong, China

**Keywords:** KRAS-mutated lung cancer, MEK inhibitor, radiation, cGAS-STING pathway, chemokine (C-X-C motif) ligand 10, immune activation

## Abstract

**Introduction:**

*Ki-ras2* (KRAS) mutation is a common driver of lung cancer, and KRAS-mutated tumors are relatively resistant to radiotherapy. Previously, we demonstrated that mitogen-activated protein kinase (MEK) inhibitors (MEKi) enhanced treatment efficacy by increasing the anti-tumor immune response after radiotherapy in KRAS-mutant tumors. In this study, we explored the potential mechanism underlying the MEKi-mediated increase in anti-tumor immune response.

**Methods and result:**

RNA sequencing revealed that the MEKi+radiotherapy combination significantly activated the chemokine signaling pathway. Blocking the C-X-C motif chemokine ligand 10 (CXCL10) receptor reduced T-cell infiltration *in vivo*. The MEKi+radiotherapy combination increased CXCL10 expression and activated the cyclic GMP–AMP synthase-stimulator of interferon genes (cGAS-STING) pathway in KRAS-mutant lung cell lines. Using a STING inhibitor and cGAS-knockout LLC cells, we showed that CXCL10 production was mediated by the cGAS-STING pathway via nuclear factor kappa B activation. This combination also increased DNA damage and apoptosis in KRAS-mutant lung cancer cell lines, triggering the cGAS-STING pathway. Western blot analysis revealed that MEKi reduced checkpoint kinase 2 phosphorylation after radiotherapy, hindering DNA repair and increasing DNA damage. Flow cytometry revealed that MEKi combined with radiotherapy boosted tumor-infiltrating CD4+ and CD8+ T cells *in vivo*, enhancing their cytotoxic and secretory functions. In an LLC-bearing mouse model, combining MEKi with varying radiotherapy doses and extending drug holidays revealed that low-dose radiotherapy with MEKi effectively controlled tumor growth.

**Conclusion:**

Our findings suggest that MEKi activates the cGAS-STING-TANK-binding kinase 1-nuclear factor kappa B-CXCL10 axis post-radiotherapy in KRAS-mutant lung cancer, increasing T-cell infiltration and function, activating anti-tumor immunity, and inhibiting tumor growth. These results indicate the potential for clinical translation.

## 1 Introduction

*Ki-ras2* (KRAS) mutations serve as driver genes in non-small cell lung cancer (NSCLC), with approximately 27% incidence among Caucasians (1) and 9% among Chinese (2). NSCLC harboring KRAS mutations is associated with a poor prognosis, and first-line platinum-based chemotherapy (± immunotherapy) is a recommended option (3). To date, five novel agents targeting KRAS have been approved by the U.S. Food and Drug Administration and the National Medical Products Administration of China (4). Nevertheless, these agents demonstrate efficacy exclusively in NSCLC with KRAS G12C mutations; moreover, their overall effectiveness remains unsatisfactory, and drug resistance occurs at 6–8 months (5–7).

Radiotherapy (RT) is a pivotal modality in cancer treatment and is known for its ability to activate anti-cancer immunity (8). RT does far more than induce DNA damage; it acts as an *in situ* vaccine that can initiate and amplify systemic anticancer immunity. Additionally, research indicates that RT can directly influence immune cells in the tumor microenvironment and modulate the expression of immunotherapy targets (9). Nevertheless, the expression of mutant KRAS influences the radiation-induced DNA damage response, leading to increased cell survival and resistance to radiation (10, 11). As mitogen-activated protein kinase kinase (MEK) is a key protein in the KRAS signaling pathway, MEK inhibitors (MEKis) can disrupt the downstream signaling cascade of KRAS. MEKis have been approved for use in *B-Raf*–mutated solid cancers, such as melanoma, colorectal cancer, and NSCLC (12). In addition to inhibiting tumor growth, our preliminary research (13) suggests that MEKis can increase chemokine secretion and increase T-cell infiltration after RT in KRAS-mutant tumors, thereby improving treatment efficacy. However, the precise mechanisms underlying this effect remain unclear.

In this study, we aimed to elucidate the mechanisms by which MEKis activate anti-tumor immunity in KRAS-mutant lung cancer. Specifically, we aimed to identify the key factors through which MEKis enhance T-cell infiltration, delineate the signaling pathways and molecular mechanisms that govern the upregulation of these factors, and comprehensively characterize the influence of the MEKis and RT combination on the functional state of infiltrating T cells. Furthermore, we investigated the impact of varying radiation doses and drug holiday schedules on therapeutic efficacy, with the intention of developing novel therapeutic strategies for patients with KRAS-mutated lung cancers.

## 2 Materials and methods

### 2.1 Tumor cell lines

NSCLC cell lines (A549 RRID: CVCL_0023 and H23 RRID: CVCL_1547) and mouse cell lines (Lewis lung carcinoma cell line [LLC] RRID: CVCL_4358) were purchased from the Cell Resource Center of the Chinese Academy of Sciences (Beijing, China). A549 has the KRAS G12S mutation, H23 has the KRAS G12C mutation, and LLC has both the KRAS G12C and NRAS Q61H mutations. A549 and H23 cells were cultured in RPMI 1640 supplemented with 10% fetal bovine serum and 1% penicillin-streptomycin solution. LLC cells were cultured in Dulbecco’s modified Eagle medium supplemented with 10% fetal bovine serum and 1% penicillin–streptomycin solution. The cyclic GMP–AMP synthase knockout (cGAS-KO) LLC cell line, along with its control, was provided by the Tongji University School of Medicine. All experiments were performed using mycoplasma-free cells.

### 2.2 Apoptosis and cell cycle analyses

To evaluate apoptosis, following the exposure of LLC and H23 tumor cells to dimethyl sulfoxide and 10 nmol/L of the MEKi trametinib for 1h, they were irradiated with a dose of 8 Gy. Similarly, A549 tumor cells were treated with dimethyl sulfoxide and 100 nmol/L trametinib for 1h, followed by irradiation at a dose of 12 Gy. At 48h post-treatment, cells from each group were trypsinized and analyzed using the Annexin V-FITC Apoptosis Detection Kit (Beyotime, Shanghai, China; Cat. No. C1062S) according to the manufacturer’s instructions.

To assess cell cycle distribution, tumor cells were subjected to the same treatment protocol as described above. After 12h, the cells were harvested via trypsinization, washed with cold phosphate-buffered saline (PBS), and fixed in 70% ice-cold ethanol overnight at −20°C. The fixed cells were washed with cold PBS and incubated with 0.5 ml of propidium iodide/RNase staining buffer (BD Biosciences, Cat. No. 550825) for 15 min at room temperature before analysis.

### 2.3 Western blot analysis

After radiation or drug treatment, the cells were harvested and lysed using radioimmunoprecipitation assay lysis buffer (Beyotime; Cat. No. P0013B) containing a protease inhibitor (Beyotime; Cat. No. ST506) and phosphatase inhibitor cocktail (APExBIO, Houston, TX, USA; Cat. No. K1015). After determining the protein concentrations of the cell lysates using the Quick Start™ Bradford Protein Assay Kit (Bio-Rad; Cat. No. 5000205), 50 μg of protein samples and 5 µl of protein marker (EpiZyme, Shanghai, China; Cat. No. WJ107) were separated on 10% or 12% sodium dodecyl sulfate-polyacrylamide gels and transferred to polyvinylidene fluoride membranes. The membranes were blocked in a 5% skim milk solution for 1h and incubated overnight with the corresponding primary antibodies at 4°C. The primary antibodies used were anti-mouse cGAS (1:1000; Cell Signaling, Danvers, MA, USA; Cat. No. 31659s), anti-extracellular signal-regulated kinase (ERK; 1:1000; Cell Signaling; Cat. No.9102), anti-phospho-ERK1/2 (Thr202/Tyr204) (1:1000; Cell Signaling; Cat. No. 4370), anti-stimulator of interferon genes (STING; 1:1000; Cell Signaling; Cat No. 13647s), anti-mouse phospho-STING (Ser365) (1:1000; Cell Signaling; Cat. No. 72971s), anti-human phospho-STING (Ser366) (1:1000; Cell Signaling; Cat. No. 50907S), anti-TANK-binding kinase 1 (TBK1; 1:1000; Cell Signaling; Cat. No. 3504s), anti-phospho-TBK1 (Ser172) (1:1000; Cell Signaling; Cat. No. 5483s), anti-interferon regulatory factor 3 (IFR3; 1:1000; Affinity Biosciences, Cincinnati, OH, USA; Cat. No. DF6895), anti-phospho- IFR3 (Ser396) (1:1000; Affinity; Cat. No. AF2436), anti-nuclear factor kappa B (NF-κB p65; 1:1000; Affinity; Cat. No. AF5006), anti-phospho- NF-κB p65 (Ser536) (1:1000; Cell Signaling; Cat. No. 3033S), anti-checkpoint kinase 1 (CHK1; 1:1000; Med Chem Express; Cat. No. HY-P80617), anti-phospho-CHK1 (Ser345) (1:1000; Cell Signaling; Cat. No. 2348S), anti-CHK2 (1:1000; Cell Signaling; Cat. No. 2662T), anti-human phospho-CHK2 (Thr68) (1:1000; Cell Signaling; Cat. No. 2661S), anti-mouse phospho-CHK2 (Thr68) (1:1000; Affinity; Cat. No. AF3036), anti-histone protein H2AX (γ-H2AX; 1:1000; Cell Signaling; Cat. No. 9718T), and anti-glyceraldehyde-3-phosphate dehydrogenase (GAPDH; 1:3000; Affinity; Cat. No. AF7021). Subsequently, the membranes were washed and incubated at 25°C with horseradish peroxidase-conjugated goat anti-rabbit for 1h. The bands were visualized using an enhanced chemiluminescence detection reagent.

### 2.4 RNA extraction and real-time quantitative polymerase chain reaction

Tumor cells were harvested, and RNA was extracted using a total RNA extraction kit (Fastagen Biotech, Shanghai, China; Cat. No. RNAfast200). Complementary DNA was synthesized from 1 µg of purified total RNA using the *Evo M-MLV* RT Master Mix (Accurate Biology, Wuhan, China; Cat. No. AG11706) according to the manufacturer’s instructions. The mRNA expression levels of C-X-C motif chemokine ligand 10 (CXCL10) in human and mouse tumor cells were quantified using specific primers. The analysis was conducted via the comparative cycle threshold method, with normalization to GAPDH expression levels. All experiments were conducted in triplicate. The primer sequences for mouse CXCL10 were as follows: forward primer ATCCGGAATCTAAGACCATCAAGAA and reverse primer TGTCCATCCATCGCAGCAC. For mouse GAPDH, the primer sequences were: forward primer GTATGACTCCACTACCGGCAAA and reverse primer GGTCTCGCTCCTGGAAGATG. For human CXCL10, the primer sequences were: forward primer TGCCATTCTGATTTGCTGCC and reverse primer TGCAGGTACAGCGTACAGTT. For human GAPDH, the primer sequences were: forward primer GTTGCCATCAATGACCCCTT and reverse primer AGAGGCAGGGATGATGTTCT.

### 2.5 Enzyme-linked immunosorbent assay

The tumor-secreted CXCL10 was quantitated from cells in 6-well plates using an ELISA Kit (Boster Biological Technology, Pleasanton, CA, USA; Cat. No. EK0736 and EK0735). Briefly, cell-free supernatants from a tumor treatment system were collected after 48h, and 100 μl/well of standards and samples were loaded into 96-well plates. After incubation with biotinylated antibodies, streptavidin-conjugated horseradish peroxidase (HRP) was added to each well and reacted with HRP substrate solution. Optical density at 450 nm was measured, and the concentration levels were adjusted according to every 10^6^ living tumor cells.

### 2.6 Ethics statement for animal experiments

Male C57BL/6 mice (4- to 6-week-old) were purchased from the Beijing SIPEIFU Biotechnology Company (Beijing, China). All animal studies were approved by the Animal Care Committee of Jinan Central Hospital (No. JNCHIACUC2024-69) and complied with the current Chinese regulations and standards for laboratory animal use. All the mice were euthanized using a carbon dioxide anesthesia protocol.

### 2.7 *In-vivo* tumor growth and mouse treatments

In total, 5 × 10^5^ LLC cells were subcutaneously injected into the right flank of C57BL/6 mice. When the tumor volume reached 50–75 cm^3^, six mice each were randomly assigned to the following three groups: control, MEKi+RT+anti-C-X-C motif chemokine receptor 3 (CXCR3), and MEKi+RT+isotype. The mice in the MEKi+RT+anti-CXCR3 and MEKi+RT+isotype groups were administered trametinib (1 mg/kg) via oral gavage daily, and those in the control group were administered the solvent (corn oil) via oral gavage at the same time. After 2 days of oral gavage, the mice in the combined therapy groups received radiation (8 Gy/1F), and 200 µg of CXCR3 neutralizing antibody (Bioxcell, Cat. No. BE0249) or the isotype (Bioxcell, Cat. No. BE0091) was injected intraperitoneally twice a week. Tumor volumes were measured every other day using a digital caliper and calculated as 0.5 × length × width^2^. When the tumors reached the size limit (2 cm^3^), the mice were euthanized, and tumors were isolated and weighed.

In a separate experimental design aimed at investigating the role of tumor-infiltrating T cells, LLC cells were similarly injected into C57BL/6 mice. Upon tumors reaching a volume of 200–250 cm³, the mice were randomly assigned to one of four groups: Control, MEKi, RT, and MEKi+RT. After 1 day of oral administration via gavage, the mice in the RT and MEKi+RT groups underwent radiation treatment (8 Gy/1F), and 3 days later, they were euthanized, and tumors were excised for further analysis.

In another experiment, LLC cells were injected into the bilateral flanks of C57BL/6 mice to assess the efficacy of a novel combination treatment modality involving MEKi and RT. Once tumor volumes attained 50–75 cm³, the mice were randomly allocated into the same four groups as previously delineated. Following one day of oral gavage, the mice in the RT and MEKi+RT groups received radiation (8 Gy/1F on the left side and 4 Gy/1F on the right side). Both the tumors and the spleen were excised for further analysis.

### 2.8 Flow cytometry analysis

Following the excision of the tumor tissue, it was rinsed with PBS to eliminate any visible adipose tissue or ecchymosis. Subsequently, the tissue was sectioned into 1 mm³ fragments and transferred into a 24-well plate. One milliliter of collagenase solution (Solarbio, Cat. No. C8160) at a concentration of 1 mg/ml was added to each well, and the samples were incubated at 37°C for 50 min, after which a filter mesh was used to separate any undigested tissue fragments. The resultant cell suspension was either collected directly or isolated using the Mouse Tumor Infiltrating Lymphocyte Isolation Kit (Solarbio, Beijing, China; Cat. No. P9000) in accordance with the manufacturer’s protocol. Flow cytometry was performed to detect mouse T cells after staining with specific marker-specific fluorescent antibodies. For intracellular staining of interferon-γ (IFN-γ), perforin and granzyme B, tumor-infiltrated immune cells, or erythrocyte-excluded spleen cells were first incubated with a cell stimulation cocktail plus protein transport inhibitors (eBioscience; Cat. No. 4975-03) at 37°C under 5% CO_2_ for 2h.

The following antibodies were used: eBioscience™ Fixable Viability Dye eFluor™ 506 (Invitrogen™; Cat. No. 65-0866-14), allophycocyanin (APC)/cyanine7 anti-mouse CD45.2 (Biolegend; Cat. No. 109824), fluorescein isothiocyanate (FITC)-anti-mouse CD3 (Biolegend, San Diego, CA, USA; Cat. No. 100203), PerCP 5.5-anti-mouse CD4 (Biolegend; Cat No. 100434), APC-anti-mouse CD8 (Biolegend; Cat. No. 100712), Brilliant Violet 421™ anti-mouse CD107a (LAMP-1) (Biolegend; Cat no. 121617), Brilliant Violet 650™ anti-mouse IFN-γ antibody (Biolegend; Cat No. 505831), phycoerythrin (PE) anti-mouse IFN-γ (Biolegend; Cat No. 505808), and PE/cyanine7 anti-human/mouse granzyme B (Biolegend; Cat No. 372214). The gating strategy for the analysis of the T cells is shown in **Supplementary Figure S1**. The stained cells were analyzed on a FACS Calibur flow cytometer (BD Bioscience), and data were analyzed using the FlowJo10 software (Tree Star, Inc., Ashland, OR, USA).

### 2.9 Immunofluorescence

Mouse cancer tissues were embedded in paraffin and sectioned into 4 μm slices. The tissue sections were subjected to deparaffinization and rehydration through a series of decreasing ethanol concentrations. Following antigen retrieval, the sections were treated with 3% hydrogen peroxide for 20 min. Subsequently, the tissue sections were incubated with the specific primary antibodies and HRP-conjugated secondary antibodies. After counterstaining the nuclei with DAPI, an autofluorescence quencher was applied, followed by mounting the sections on slides and acquiring the images. The primary antibodies used included anti-CD4 (1:2000; Abcam, Cambridge, UK; Cat No. ab183685), anti-CD8 (1:2000; Abcam; Cat No. ab237723), and anti-CXCL10 (1:2000; Proteintech, Rosemont, IL, USA; Cat No. 10937-1-AP). Cells subjected to the corresponding treatments were seeded onto chamber slides. Following fixation and blocking, the slides were incubated with anti-γ-H2AX (1:500; Cell Signaling; Cat No. 9718T), and the subsequent procedures were conducted as previously described.

### 2.10 Gene sequencing

Twenty-four hours after the designated treatment, LLC cells cultured in 60 mm dishes in the four groups were subjected to two rinses with PBS and treated with 1 ml of TRIzol reagent each. The solution was pipetted until it appeared clear, after which the mixture was transferred to an EP tube and stored at −80°C. The samples were then transported to Beijing Genomics Institute on dry ice for gene sequencing. Sequencing commenced once the samples successfully passed quality control assessments.

### 2.11 Statistical methods

All data, unless mentioned otherwise, are represented as the mean ± standard deviation (SD). Statistical analysis was performed using SPSS 24 (IBM SPSS Statistics for Windows, Version 24.0. Armonk, NY: IBM Corp.) and GraphPad Prism 8.0 (GraphPad Software, San Diego, CA, USA). At least three independent experiments were conducted. Inter-group comparisons were conducted utilizing a one-way analysis of variance, followed by Tukey’s *post-hoc* test to address multiple pairwise comparisons. The differences were considered statistically significant at *P* < 0.05.

## 3 Results

### 3.1 CXCL10 is a critical factor in KRAS-mutated lung cancer treated with MEKi+RT

**Figure 1:**
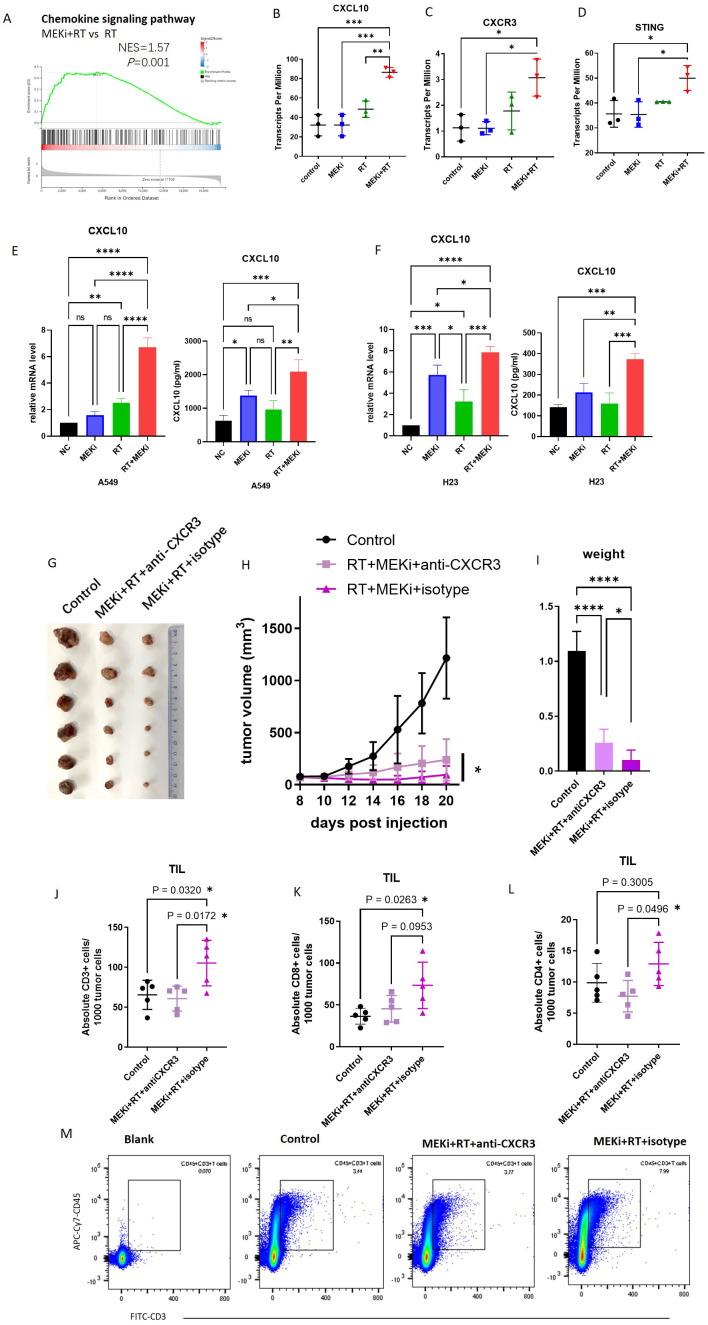
CXCL10 is a critical factor in KRAS-mutated lung cancer treated with MEKi+RT. **(A)** Tissue sequencing reveals enrichment of the chemokine signaling pathway. **(B-D)** Sequencing data indicate CXCL10, CXCR3, and STING expression across four groups. **(E)** CXCL10 mRNA and protein levels in A549 post-MEKi and RT treatment. **(F)** CXCL10 mRNA and protein levels in H23 post-MEKi and RT treatment. **(G)** Mice injected with LLC cells were categorized into control, MEKi+ RT+anti-CXCR3, and MEKi+RT+isotype groups, with the tumor sizes measured. **(H)** Tumor growth curves for the three groups. **(I)** Tumor weight comparison post-excision. **(J-L)** Flow cytometry comparison of CD3+, CD8+, and CD4+ T lymphocyte counts in tumors. **(M)** Flow cytometry plots showing CD3+ T cell gating in the three groups. *P < 0.05; **P < 0.01; ***P < 0.001; ****P < 0.0001. CXCL, C-X-C motif chemokine ligand; CXCR, C-X-C motif chemokine receptor; MEKi, MEK inhibitor; RT, radiotherapy; STING, stimulator of interferon genes.

A comprehensive analysis of tissue sequencing data from mouse LLC tumors previously subjected to MEKi and/or RT revealed significant enrichment of the chemokine signaling pathway in the MEKi+RT treatment group (**Figures 1A**), which included a marked upregulation of CXCL10 and its receptor CXCR3 (**Figures 1B, C**), whereas other cytokines, including CXCL9, C-C motif chemokine ligand (CCL)1, and CCL2, did not exhibit statistically significant increases in the combination group (**Supplementary Figure S2**). This suggests a potentially critical role for CXCL10 in the therapeutic efficacy of the MEKi and RT combination therapy. Additionally, elevated CXCL10 expression was observed in two human KRAS-mutant lung cancer cell lines, A549 and H23 (**Figures 1E, F**), in the combination group, similar to that in LLC (13).

To assess the functional impact of CXCL10, the use of an *in-vivo* neutralizing antibody against CXCR3 revealed that CXCR3 neutralization attenuated the tumor control efficacy of the MEKi and RT combination therapy, as evidenced by increased tumor volume and weight (**Figures 1G–I**). Flow cytometric analysis of tumor-infiltrating lymphocytes indicated a reduction in the number of infiltrating CD3, CD8, and CD4 T lymphocytes in the MEKi+RT+anti-CXCR3 group compared to the MEKi+RT+isotype group (**Figures 1J–M**).

### 3.2 CXCL10 induction is associated with the cGAS-STING signaling pathway after MEKi+RT treatment

**Figure 2:**
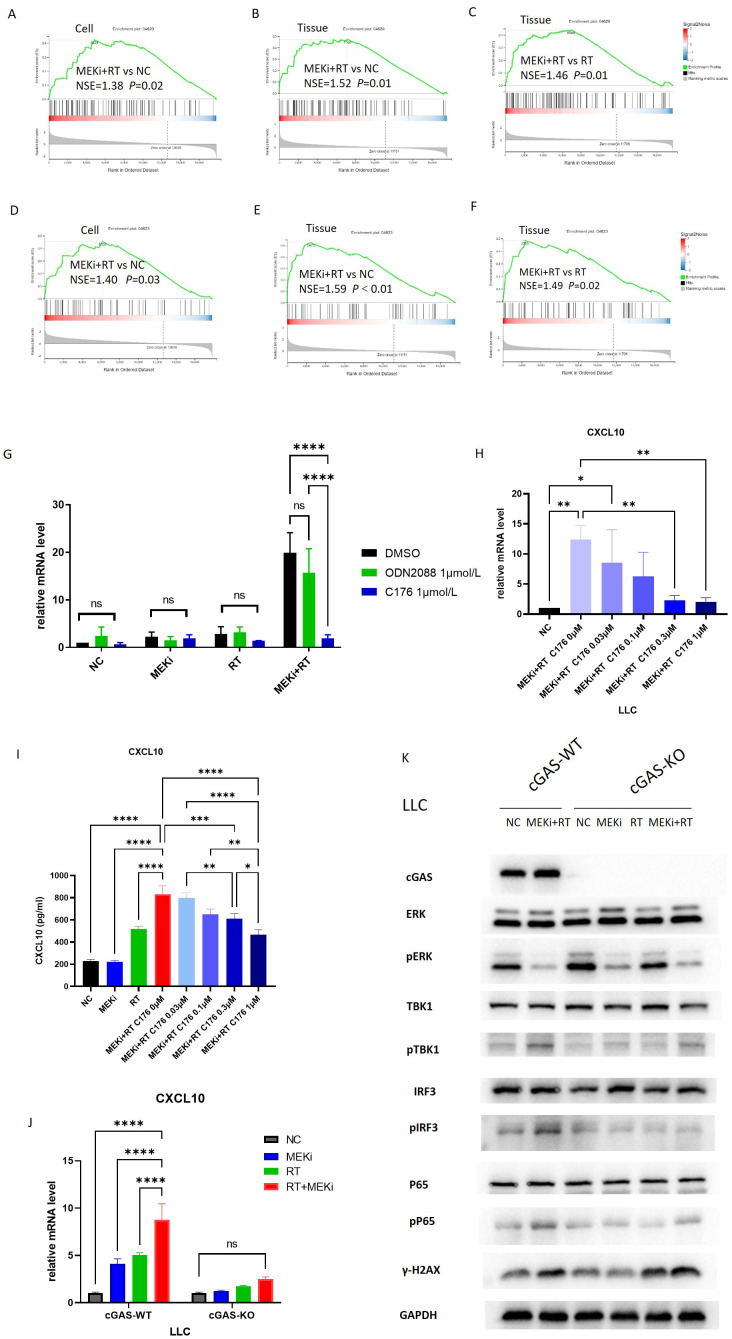
The cGAS-STING signaling pathway is associated with CXCL10 induction after MEKi+RT treatment. **(A–C)** Sequencing data shows enrichment of Toll-like receptor pathway. **(D–F)** Sequencing data indicates cytosolic DNA-sensing pathway enrichment. **(G)** PCR analysis of CXCL10 in LLC cells treated with TLR inhibitor (1 µmol/L) and STING inhibitor C176 (1 µmol/L). **(H)** Impact of varying C176 concentrations on CXCL10 mRNA expression in LLC cells treated with MEKi and RT. **(I)** Effect of C176 concentrations on CXCL10 protein levels in LLC cell culture supernatant after MEKi and RT treatment. **(J)** CXCL10 mRNA expression in cGAS-WT and cGAS-KO LLC cell lines across four treatment groups. **(K)** Western blot analysis of cGAS-STING pathway activation in cGAS-WT and cGAS-KO LLC cell lines. *P < 0.05; **P < 0.01; ***P < 0.001; ****P < 0.0001. cGAS, cyclic GMP–AMP synthase; CXCL, C-X-C motif chemokine ligand; KO, knock-out; MEKi, MEK inhibitor; STING, stimulator of interferon genes; RT, radiotherapy; WT, wild-type.

Radiation-induced DNA damage is the main trigger for immune activation (14). Sequencing analysis of LLC cells and tissues treated with MEKi alone, RT alone, or MEKi+RT revealed significant enrichment of the Toll-like receptor signaling (**Figures 2A–C**) and cytosolic DNA-sensing pathways (**Figures 2D–F**), and STING expression (**Figure 1D**) in the MEKi+RT group. The Toll-like receptor family member, Toll-like receptor 9, and the cGAS-STING DNA-sensing pathway are capable of detecting intracellular DNA damage, thereby inducing immune activation (15–17). In LLC cells, the STING protein inhibitor, C-176, effectively inhibited CXCL10 production in the MEKi+RT group, whereas the TLR9 receptor inhibitor, ODN2088, did not (**Figure 2G**). Furthermore, a concentration-dependent effect of C-176 was observed at both the mRNA and protein levels of CXCL10 in the MEKi+RT group (**Figures 2H, I**).

To further elucidate the role of the cGAS-STING pathway, cGAS-KO LLC cells were subjected to the same treatment as above, that is, treatment with MEKi alone, RT alone, or MEKi+RT. MEKi+RT failed to effectively induce CXCL10 expression in cGAS-KO cells (**Figure 2J**). Moreover, the expression levels of the downstream molecules in the cGAS-STING pathway, such as pTBK1, phosphorylated or activated form of the protein interferon regulatory factor 3 (pIRF3), and human cytomegalovirus phosphoprotein 65 (pP65), were reduced, whereas that of the DNA damage marker γ-H2AX remained unchanged (**Figure 2K**).

### 3.3 MEKi+RT treatment activated the cGAS-STING signaling pathway

**Figure 3:**
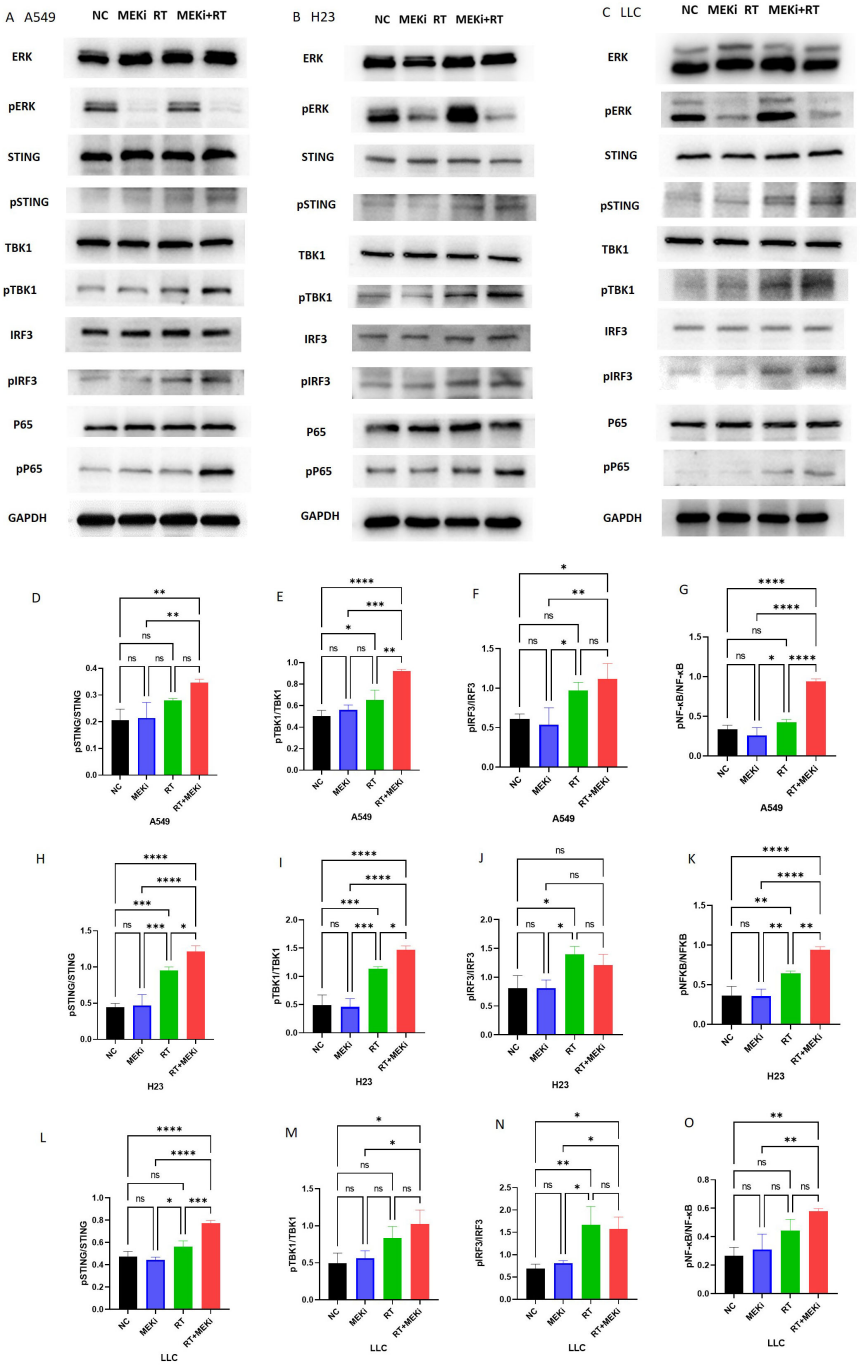
Activation of the cGAS-STING pathway proteins by MEKi+RT. **(A–C)** Western blot analysis of the cGAS-STING pathway activation in A549, H23, and LLC cell lines, respectively. **(D–O)** Statistical analysis of pSTING, pTBK1, pIFR3, and pP65 in A549, H23, and LLC cell lines, respectively. *P < 0.05; **P < 0.01; ***P < 0.001; ****P < 0.0001. cGAS-STING, cyclic GMP–AMP synthase- stimulator of interferon genes; MEKi, MEK inhibitor; pIFR3, phosphorylated interferon regulatory factor 3; pTBK, phosphorylated TANK-binding kinase 1; RT, radiotherapy.

After treatment with MEKi+RT, the cGAS-STING signaling pathway was effectively activated in KRAS-mutant A549, H23, and LLC cells, with a notable increase in the expression of downstream signaling molecules, specifically phosphorylated STING and phosphorylated TBK1 (**Figures 3A–C**). However, pIRF3 expression was not significantly different between the MEKi+RT and RT groups. In contrast, the expression of phosphorylated NF-κB p65 was significantly elevated after MEKi+RT treatment (**Figures 3D–O**). Previous studies have established that NF-κB functions as a transcription factor for the expression of inflammatory cytokines that are activated downstream of the cGAS-STING pathway (18, 19). Thus, the combination of MEKi and RT may effectively activate the downstream NF-κB transcription factor in KRAS-mutant lung cancers.

### 3.4 MEKi+RT treatment increased DNA damage and inhibited DNA repair in KRAS-mutated lung cancer

**Figure 4:**
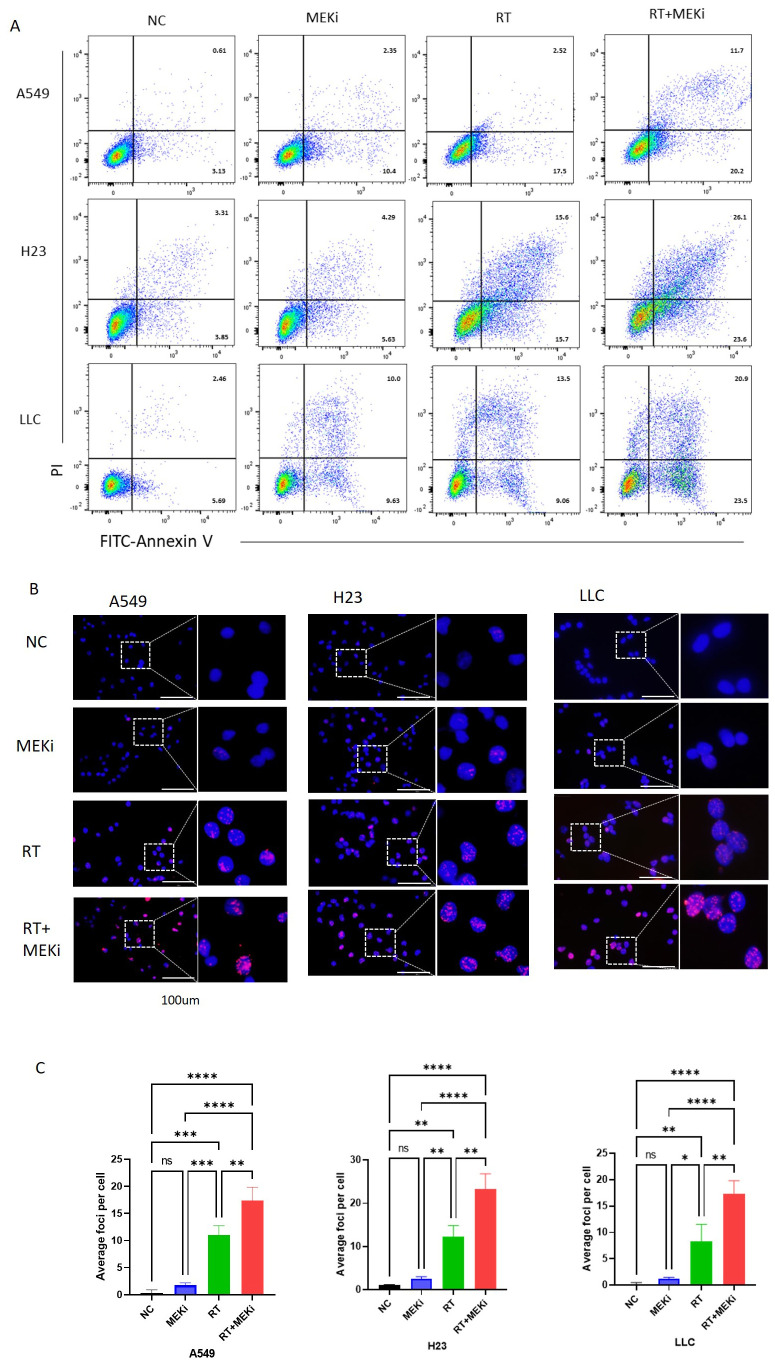
MEKi+RT increased apoptosis and DNA damage in KRAS-mutated lung cancer. **(A)** Flow cytometry analysis of apoptosis in A549, H23, and LLC cells post-treatment. **(B)** Immunofluorescence of γ-H2AX expression in these cells post-treatment. **(C)** Statistical analysis of γ-H2AX foci per cell. *P < 0.05; **P < 0.01; ***P < 0.001; ****P < 0.0001. γ-H2AX, histone protein H2AX; MEKi, MEK inhibitor; RT, radiotherapy.

The potential of MEKi to enhance post-radiation DNA damage and its underlying mechanism was explored. Flow cytometry analysis revealed that MEKi+RT significantly increased apoptosis in KRAS-mutant tumors compared to RT alone (**Figure 4A**). Immunofluorescence staining demonstrated a significant increase in the level of DNA damage marker γ-H2AX in the MEKi+RT group (**Figures 4B, C**). Cell cycle analysis revealed that MEKi plus RT combination treatment increased the proportion of the G2/M phase in H23 and A549 cells (**Supplementary Figures S3A, B**).

**Figure 5:**
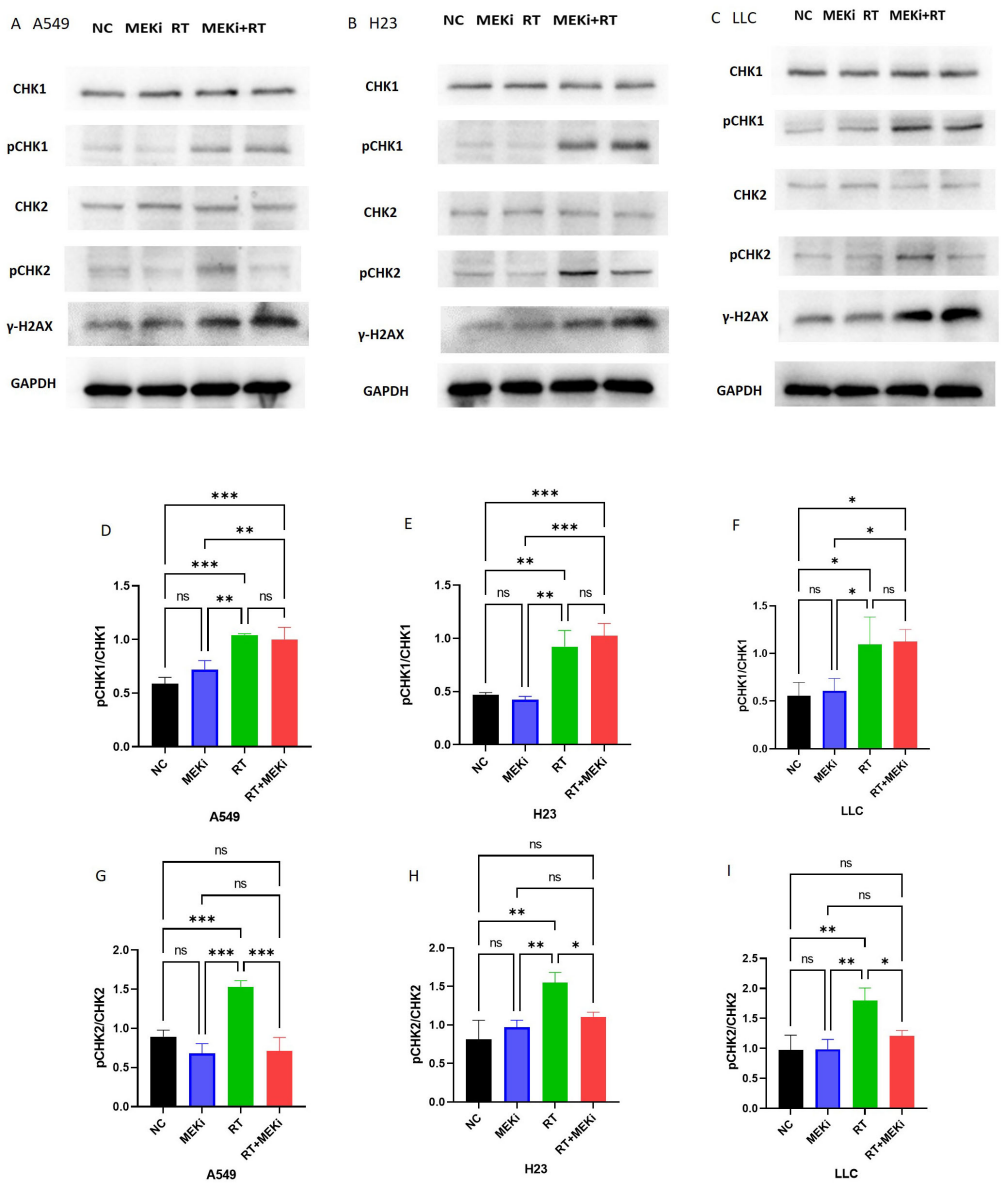
MEKi is dependent on CHK2 to inhibit repair of RT-induced DNA damage. **(A–C)** Western blot analysis of proteins related to DNA damage repair and γ-H2AX in A549, H23, and LLC cell lines, respectively. **(D–I)** Statistical analysis of pCHK1 and pCHK2 in A549, H23, and LLC cell lines, respectively. *P < 0.05; **P < 0.01; ***P < 0.001; ****P < 0.0001. CHK2, checkpoint kinase 2; γ-H2AX, histone protein H2AX; MEKi, MEK inhibitor; RT, radiotherapy.

To elucidate the mechanisms by which MEKi enhances DNA damage, the DNA damage repair pathways in tumors were investigated. Expression of the downstream markers pCHK1 and pCHK2 was significantly elevated after RT. Notably, RT+MEKi suppressed the increase in pCHK2 expression, thereby inhibiting DNA damage repair and augmenting the expression of γ-H2AX (**Figures 5A–C**). A consistent pattern was observed across the three KRAS-mutant lung cancer cell lines. Statistical analysis corroborated that the MEKi+RT effectively reduced RT-induced pCHK2 expression, while exerting no significant effect on pCHK1 (**Figures 5D–I**).

### 3.5 MEKi+RT improved the function of infiltrated T cells

**Figure 6:**
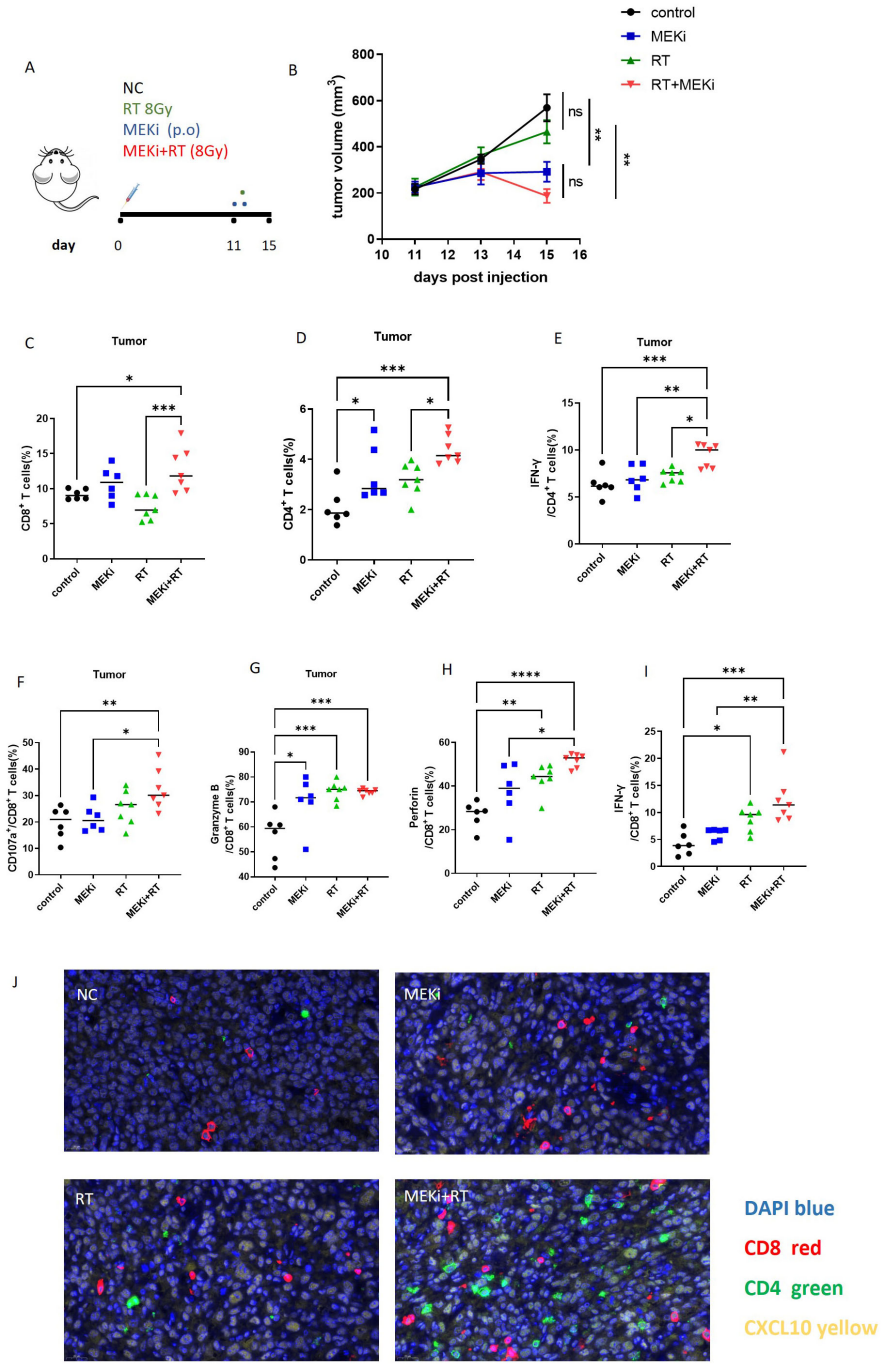
MEKi+RT improved the function of infiltrated T cells. **(A)** Diagram of treatment procedures after LLC cell inoculation in mice. **(B)** Tumor growth curves for four groups. **(C, D)** Flow cytometry analysis of the CD8+ and CD4+ T lymphocyte proportions in infiltrating immune cells. **(E–I)** Flow cytometry analysis of IFN-γ+ CD4+ T lymphocytes and CD107a+, granzyme B, perforin, and IFN-γ+ CD8+ T lymphocytes. **(J)** Immunofluorescence of CD8, CD4 T lymphocytes, and CXCL10 in tumor-infiltrating cells across groups. *P < 0.05; **P < 0.01; ***P < 0.001; ****P < 0.0001. IFN, interferon; MEK inhibitor; RT, radiotherapy.

Previously (13), we demonstrated that the combination of MEKi and RT effectively controlled tumor growth and enhanced T-cell infiltration in KRAS-mutated tumor-bearing mice. Based on these findings, an LLC treatment model was used to investigate the effect of MEKi+RT treatment on the functionality of tumor-infiltrating T cells (**Figure 6A**). Consistent with our earlier study, the treatment resulted in a similar trend of tumor reduction (**Figure 6B**). Flow cytometric analysis indicated a significant increase in the number of tumor-infiltrating CD4+ and CD8+ T cells in the MEKi+RT group (**Figures 6C, D**). Furthermore, the proportions of IFN-γ+ CD4+ T cells, as well as CD107a+, granzyme B+, perforin+, and IFN-γ+ CD8+ T cells—markers indicative of T-cell functionality—were elevated in the MEKi+RT group (**Figures 6E–I**). Additionally, multiplex immunofluorescence staining revealed enhanced infiltration of CD4+ and CD8+ T cells and the upregulation of CXCL10 expression in the MEKi+RT group (**Figure 6J**).

### 3.6 The therapeutic model using low-dose radiation in mice bearing the LLC

**Figure 7:**
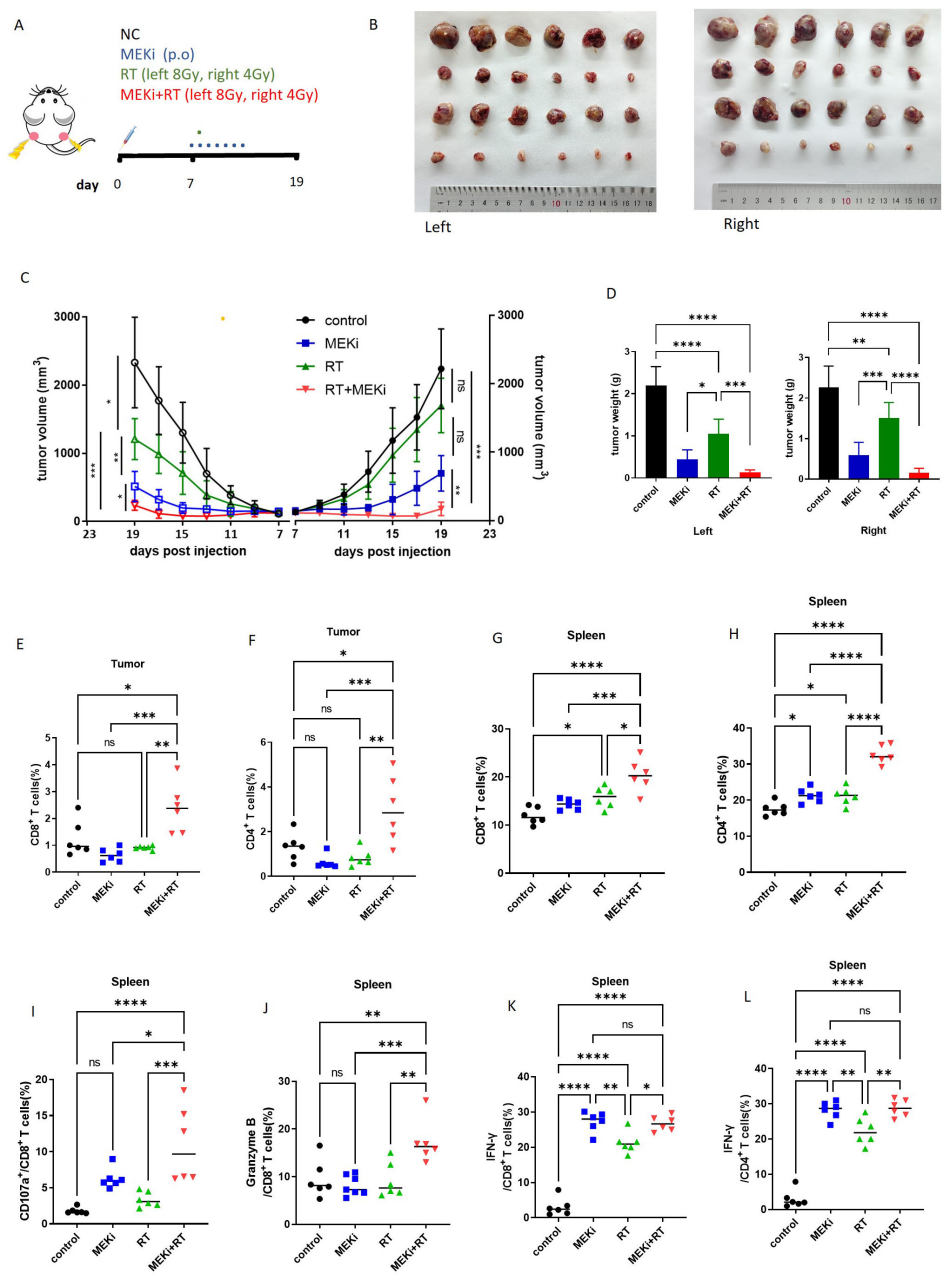
The treatment model with low-dose radiation in the mouse model. **(A)** Diagram of treatment procedures after LLC cell inoculation in mice. **(B)** Mice injected with LLC cells were categorized into control, MEKi, RT, and MEKi+RT groups, with tumor sizes measured on the left and right side. **(C)** Tumor growth curves for four groups. **(D)** Tumor weight comparison post-excision. **(E, F)** Flow cytometry analysis of CD8+ and CD4+ T lymphocyte proportions in infiltrating immune cells of the tumor on the left side. **(G, H)** Flow cytometry analysis of CD8+ and CD4+ T lymphocyte proportions in spleen cells. **(I–L)** Flow cytometry analysis of CD107a+, granzyme B, and IFN-γ+ CD8+ T lymphocytes and IFN-γ+ CD4+ T lymphocytes. *P < 0.05; **P < 0.01; ***P < 0.001; ****P < 0.0001. IFN, interferon; MEK inhibitor; RT, radiotherapy.

We further investigated whether a reduction in the duration of MEKi treatment in conjunction with low-dose radiation applied to the contralateral side could effectively elicit an immune response. The LLC treatment model was established using the specific radiation and treatment schedule detailed in **Figure 7A**. A decrease in radiation dosage resulted in an increase in tumor volume on the right side in the RT group, whereas both the left and right tumors were effectively controlled in the MEKi+RT group (**Figures 7B, C**). The infiltration of CD4+ and CD8+ T cells into the tumors on the left side was significantly higher in the MEKi+RT group than in the other three groups (**Figures 7D, E**). However, except for CD107a, no significant differences in other T-cell functional indicators were observed among the four groups (**Supplementary Figures 3C–F**). The proportions of CD4+ and CD8+ T cells in spleen samples were elevated in the MEKi+RT group relative to those in the other groups (**Figures 7G, H**). Additionally, the proportions of IFN-γ+ CD4+ T cells, CD107a+, granzyme B+, and IFN-γ+ CD8+ T cells, which serve as indicators of T-cell functionality, were also increased in the MEKi+RT group (**Figures 7I–L**).

## 4 Discussion

**Figure 8:**
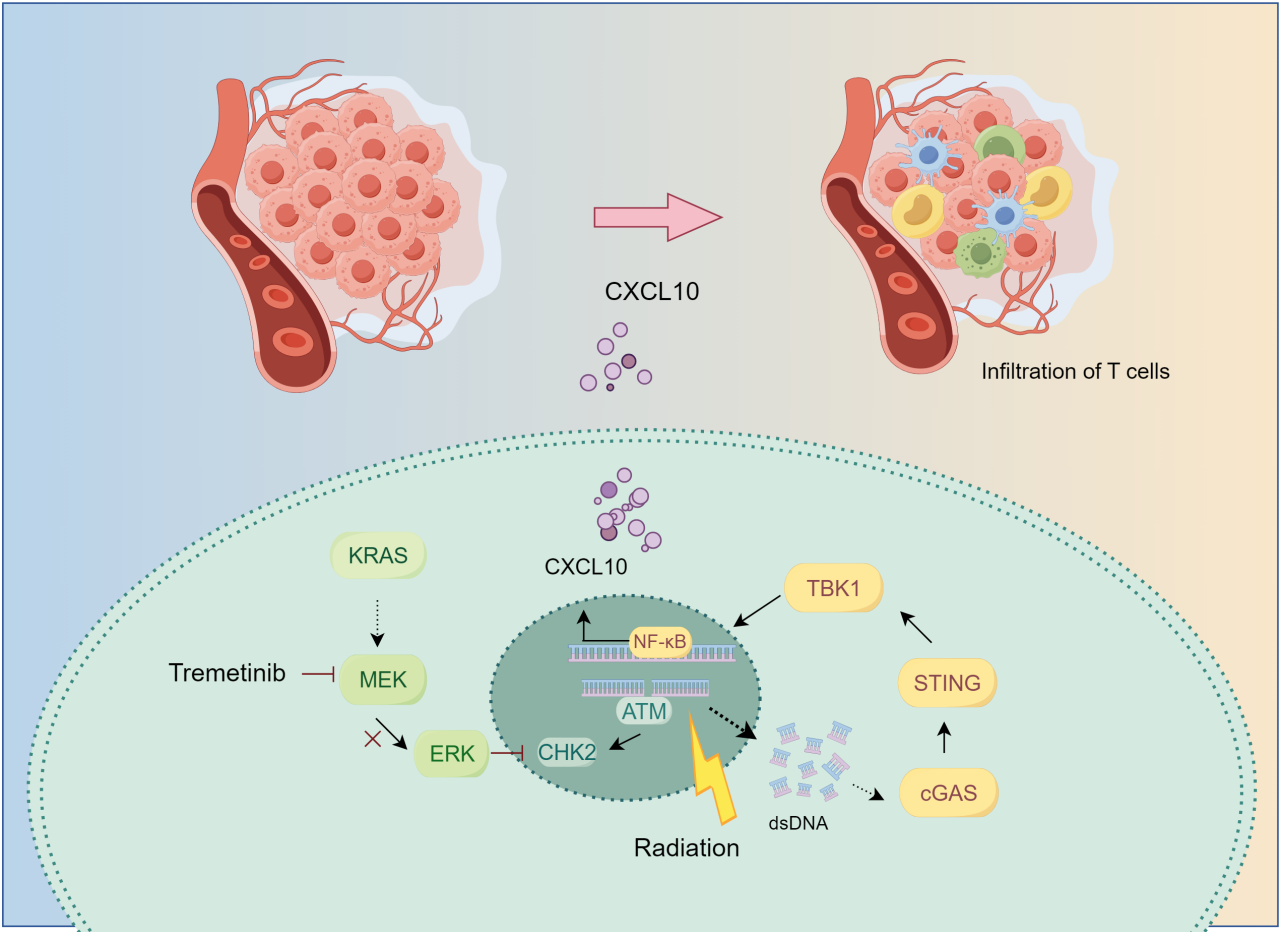
The mechanism underlying MEKi+RT treatment-mediated activation of anti-tumor immunity. In KRAS-mutated lung cancer, the administration of trametinib in combination with RT results in the inhibition of the downstream MEK-ERK signaling pathway, impacts CHK2 phosphorylation, and induces increased DNA damage. Consequently, the generation of additional double-stranded DNA may facilitate the activation of the cGAS-STING pathway. This activation stimulates the transcription factor NF-κB, leading to an upregulation of CXCL10 expression. CXCL10 promotes the infiltration of T cells into the tumor microenvironment, thereby enhancing anti-tumor immunity. CHK2, checkpoint kinase 2; cGAS-STING, cyclic GMP–AMP synthase-stimulator of interferon genes; CXCL, C-X-C motif chemokine ligand; MEK inhibitor; RT, radiotherapy.

KRAS-mutant lung cancer presents a therapeutic challenge for which novel drugs and treatment strategies are being developed (20). Although inhibition of the KRAS-MEK-ERK signaling pathway has been reported to activate the immune system (21–23), its synergistic potential with RT has not been sufficiently investigated. Previously (13), we demonstrated the efficacy of tumor control by the combination of MEKi and RT in KRAS-mutated tumors. In the current study, we found that, in KRAS-mutant NSCLC, MEKi enhanced double-stranded DNA levels by influencing RT-induced DNA damage, thereby activating the cGAS-STING pathway. This activation leads to the production of CXCL10 by tumor cells, which subsequently facilitates T-cell activation and migration toward the tumor, thereby augmenting anti-tumor immunity (**Figure 8**).

Chemokines are integral to the recruitment and maintenance of the functional capacity of T cells (24). Previously (13), we demonstrated that MEKis enhanced immune activation after RT and increased the number of tumor-infiltrating lymphocytes; moreover, several cytokines affecting tumor lymphatic infiltration were detected in MEKi and RT-treated KRAS-mutated cell lines. The expression of three chemokines, CXCL9/10/11, was significantly increased. Tissue sequencing data revealed significant enrichment of the chemokine in the LLC tumor tissues subjected to the MEKi+RT treatment. A total of 188 genes were enriched in this pathway, including CXCL8-CXCR1/2, CXCL12-CXCR4, CCL1- C-C motif chemokine receptor 8, and CXCL9/CXCL10-CXCR3. Subsequent screening revealed significant upregulation of CXCL10 and its receptor CXCR3 in the MEKi+RT group. CXCL10 predominantly targets T cells, and its interaction with the CXCR3 receptor on T-cell surfaces promotes its recruitment to tumor sites, thereby augmenting anti-tumor immunity (25, 26). Previously, we corroborated significantly enhanced CXCL10 expression in LLC cells treated with MEKi+RT. In this study, this phenomenon was further confirmed in two human KRAS-mutant cell lines. In *in-vivo* experiments, we used a blocking antibody against CXCR3 to demonstrate a reduction in the tumor control efficacy of the MEKi+RT combination treatment, which, in turn, was associated with decreased T-cell infiltration. These results indicate that the CXCL10-CXCR3 axis is crucial for mediating the therapeutic effects MEKi+RT combination treatment in managing KRAS-mutant tumors.

DNA damage resulting from RT is recognized as the primary factor initiating post-irradiation immune responses (27, 28). Our study demonstrated that the STING protein inhibitor, C-176, effectively suppressed CXCL10 production in the combined treatment group. Additionally, we observed a significant increase in phosphorylated STING and phosphorylated TBK1 levels in the MEKi+RT group across the three KRAS-mutant lung cancer cell lines. cGAS-KO LLC cells were used to confirm the induction of CXCL10 expression. Taken together, these results suggest that the combination of MEKi and RT enhances CXCL10 production by activating the cGAS-STING signaling pathway.

The cGAS-STING signaling pathway can remodel the RT-induced tumor immune microenvironment (29) and induce type I IFNs to activate immune responses, resulting in the cellular production of proinflammatory cytokines (30). However, our results revealed no enrichment in type I IFN signaling in the MEKi+RT group (data not shown), and no significant elevation in IFN-α and IFN-β levels (13). Further examination of transcription factors downstream of cGAS revealed a marked increase in pNF-κB but not pIRF3 in the combined treatment group. Based on existing literature (18, 19, 31), we hypothesize that CXCL10 expression is directly mediated via the activation of NF-κB downstream of the cGAS-STING pathway.

DNA strand breaks are lethal lesions induced by ionizing radiation that can trigger a series of cellular DNA damage responses, including those that help cells recover from radiation injuries, such as cell cycle arrest and DNA repair (32). MEKi treatment increased the level of DNA damage marker γ-H2AX, increased the proportion of G2/M-phase arrested cells, and promoted apoptosis following RT. Ataxia telangiectasia mutated- and Rad3-related-Chk1 (ATR-CHK1) and ataxia telangiectasia-mutated-CHK2 (ATM-CHK2) are the main DNA damage repair pathways that prevent cell death (32, 33). MEKi can suppress the expression and activation of several DNA damage repair proteins following RT, thereby enhancing radiosensitivity in pancreatic cancer (34). Our results revealed that MEKi effectively inhibited CHK2 phosphorylation after RT. Dai et al. reported an interaction between ERK and CHK2, and the ability of an ERK inhibitor to modulate CHK2 activity in diffuse large B-cell lymphoma (35). Therefore, MEKi may enhance double-strand breaks after RT in a CHK2-dependent manner, which further augments the accumulation of double-strand DNA within tumor cells, thereby effectively activating the cGAS-STING pathway.

The immune function status of tumor-infiltrating T lymphocytes after treatment with MEKi and RT was thoroughly analyzed. Our findings showed increased infiltration of CD4+ and CD8+ T cells, along with a higher expression of CD107a, IFN-γ, perforin, and granzyme B, indicating enhanced T-cell activation and cytotoxic function (36, 37). Nevertheless, the PACIFIC-2 study, yielding negative outcomes for the combination of immunotherapy and RT, indicates the complexity of immune activation after RT (38). Preclinical studies have demonstrated that alongside hypofractionated RT, low-dose RT induces immune activation (39). However, findings from a randomized controlled phase 2 clinical trial indicated that single-target RT, whether combined with low- or high-dose RT, did not outperform immunotherapy alone (40). A clinical trial by Zhou et al. showed that the combination of low-dose RT and hypofractionated RT for multiple lesions exhibited a favorable therapeutic effect when used in conjunction with sintilimab in treatment-naive patients with metastatic NSCLC (41). Therefore, we developed a novel anti-tumor treatment model by administering 8 Gy RT to the left side and 4 Gy low-dose RT to the right side, combined with MEKi treatment for 1 week, followed by a drug holiday. The results demonstrated effective tumor control in the MEKi+RT group, even with 4 Gy RT on the right side, with controlled tumor growth to some extent after cessation of MEKi. The enhanced functionality of splenic T cells observed in the combination group suggests that the administration of MEKi in conjunction with RT has the potential to activate immune responses. Additionally, implementing intermittent drug holidays may represent a viable strategy for managing tumor growth in the context of MEKi and RT combination therapy while simultaneously reducing the toxic side effects associated with MEKi administration in clinical practice. Similarly, Dong et al. (42) demonstrated that the intermittent administration of MEKi can delay the onset of tumor resistance. Our study also contributes to this treatment model by providing insights into KRAS-mutant tumors.

The integration of MEKis with chemotherapy, immune checkpoint inhibitors, epidermal growth factor receptor-tyrosine kinase inhibitors, and B-Raf inhibitors to enhance clinical efficacy in NSCLC has been investigated (43). Our study demonstrated the potential of RT incorporated with MEK inhibition to overcome the limitations of MEKis, which typically induce only a temporary shrinkage of KRAS-mutated lung tumors, thereby delaying the onset of drug resistance. It is a limitation that we did not directly knockdown CHK2 to further confirm the relationship. In the future, further experiments to verify the relationship between the downregulation of phosphorylated CHK2 and activation of the cGAS STING pathway, response experiments (such as overexpression of CHK2 in the context of MEKi + RT), the rescue effects on DNA damage (γ-H2AX), cGAS STING pathway activation, and CXCL10 production will be conducted to establish a more solid causal chain. Recent advancements in RT techniques, such as proton RT, flash technology, and lattice therapy, have significantly expanded the applicability of RT (44, 45). RT can now be safely administered in conditions previously deemed contraindicated. This combined approach can serve as a valuable framework for future translational studies and clinical trials.

## 5 Conclusion

MEKis upregulated CXCL10 expression after RT in KRAS-mutated lung cancer, with CXCL10 serving as a pivotal factor in immune activation. The proposed underlying mechanism involves MEKi-mediated inhibition of DNA damage repair post-RT, which robustly activates the cGAS-STING-TBK1-NF-κB-CXCL10 signaling pathway. This activation enhances tumor infiltration by T cells and improves T-cell functionality. The strategic combination of MEKis with varying doses of RT offers a more comprehensive approach to tumor control, presenting substantial promise for clinical application.

## Data Availability

The datasets presented in this study can be found in online repositories. The names of the repository/repositories and accession number(s) can be found in the article/Supplementary Material. The RNA-seq data has been uploaded to the GEO repository. (https://www.ncbi.nlm.nih.gov/geo/query/acc.cgi?acc=GSE297753, and https://www.ncbi.nlm.nih.gov/geo/query/acc.cgi?acc=GSE209767).
