# Supplementary figures and images for: Impact of MEK inhibition on T-cell infiltration and function after radiotherapy in KRAS-mutant lung cancer
Source: Front Immunol. 2025 Nov 24;16:1663502. doi: 10.3389/fimmu.2025.1663502 (PMC12682748; doi:10.3389/fimmu.2025.1663502)

Figure 2K

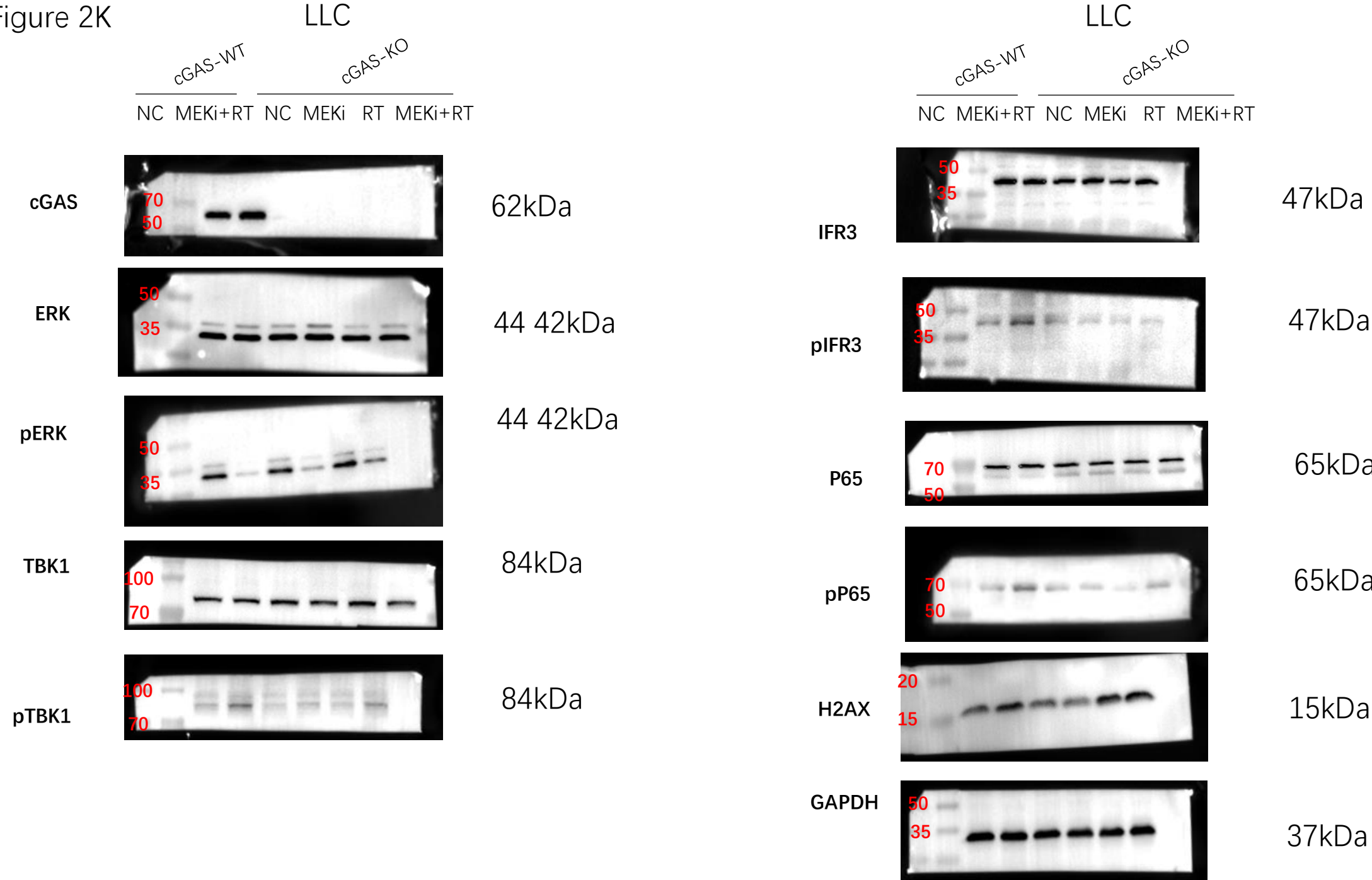

Figure 3A

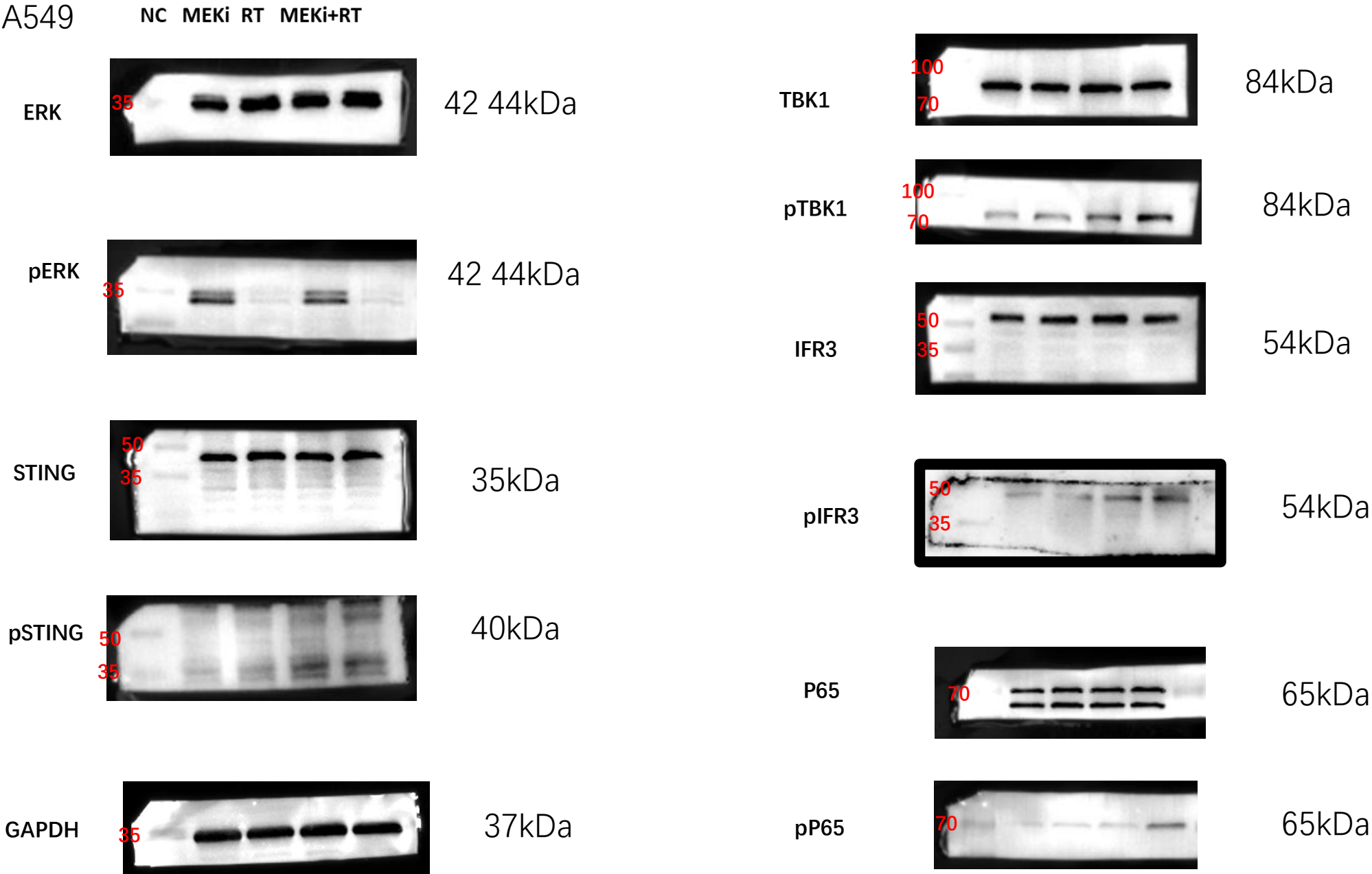

Figure 3B

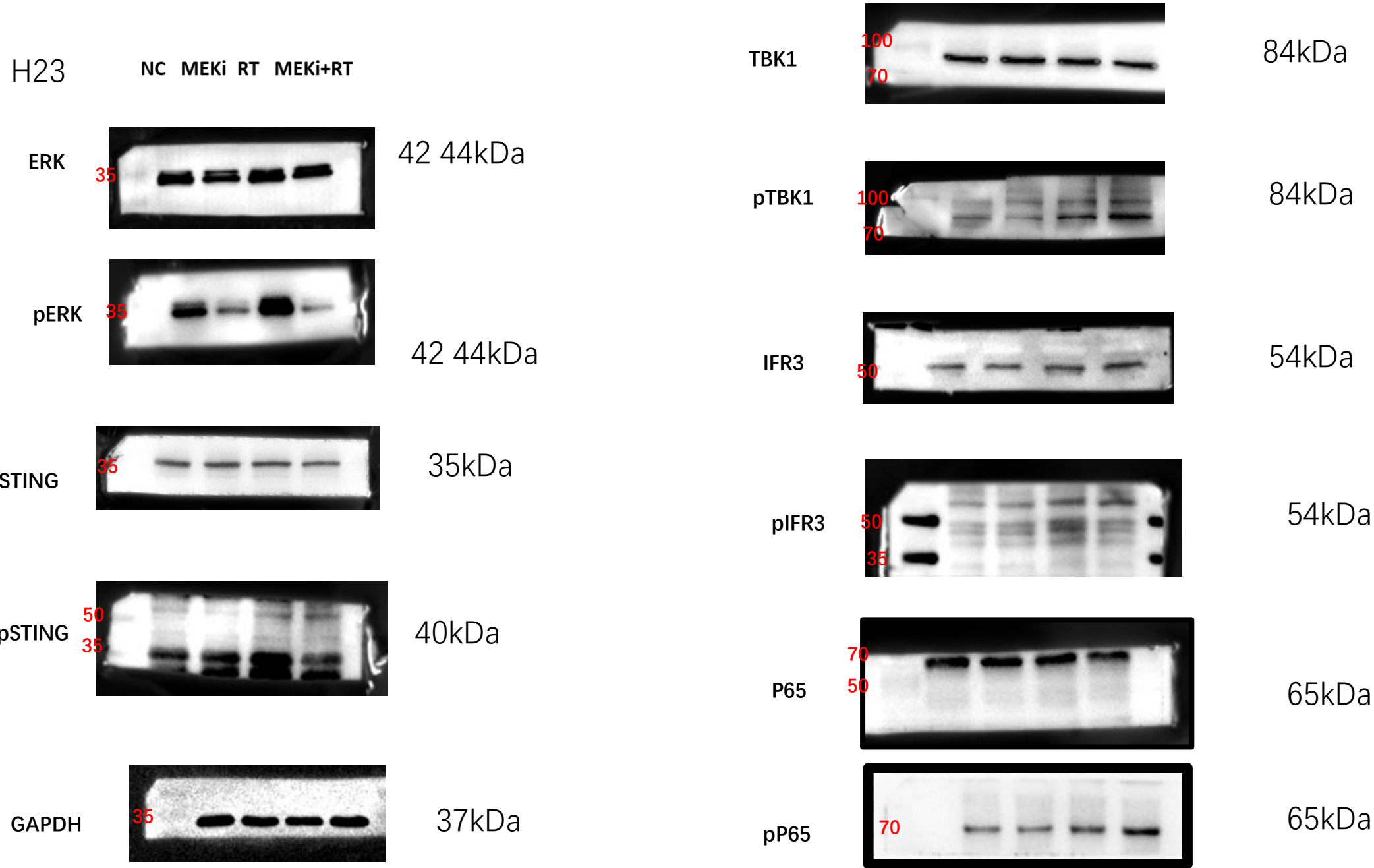

Figure 3C

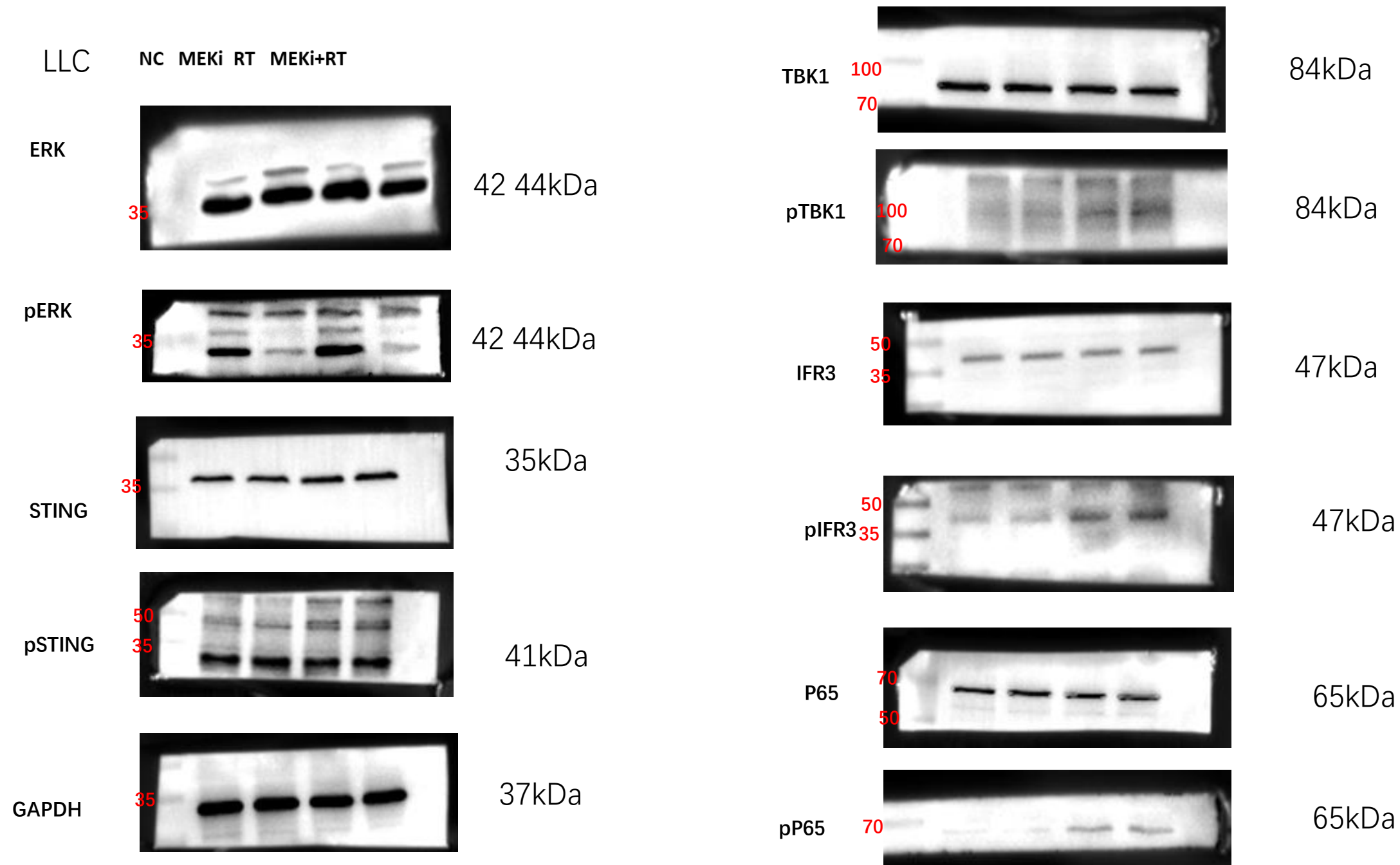

Figure 5A

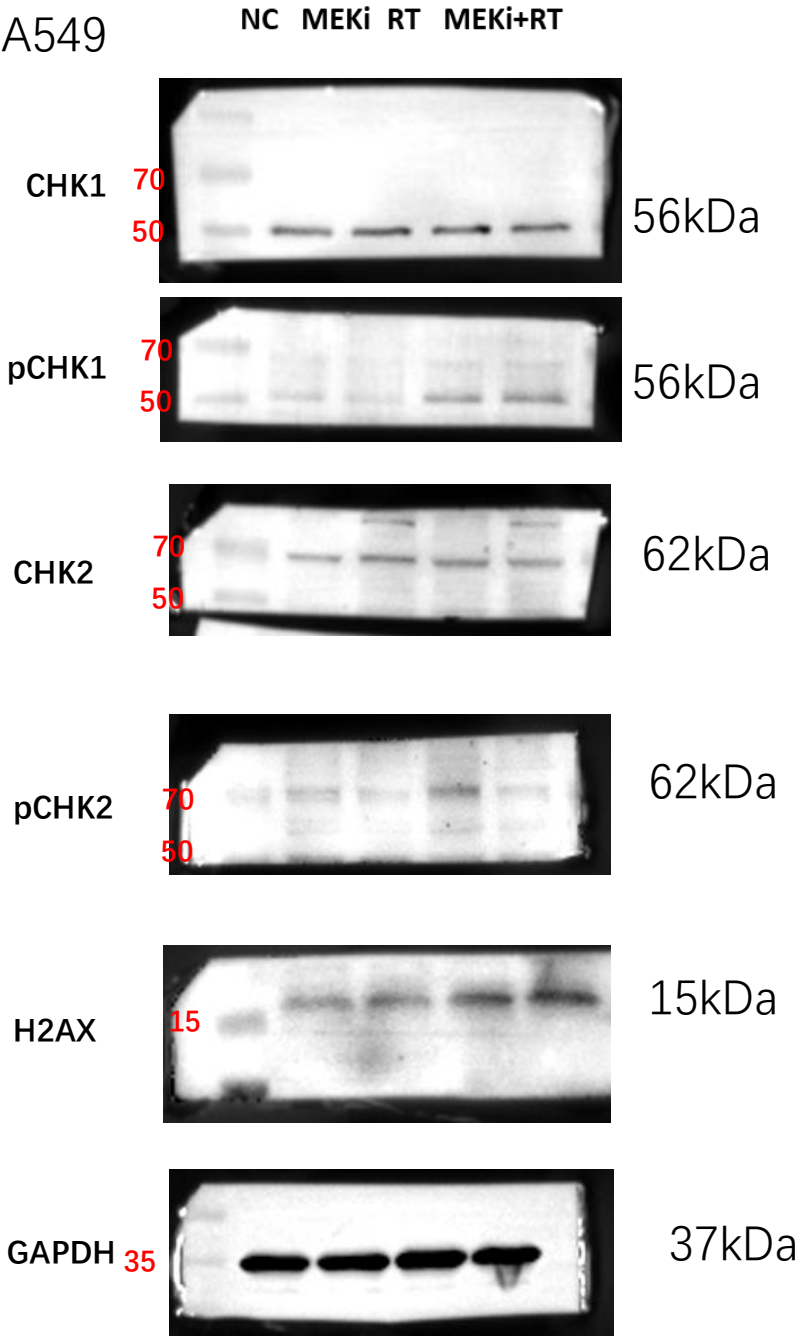

Figure 5B

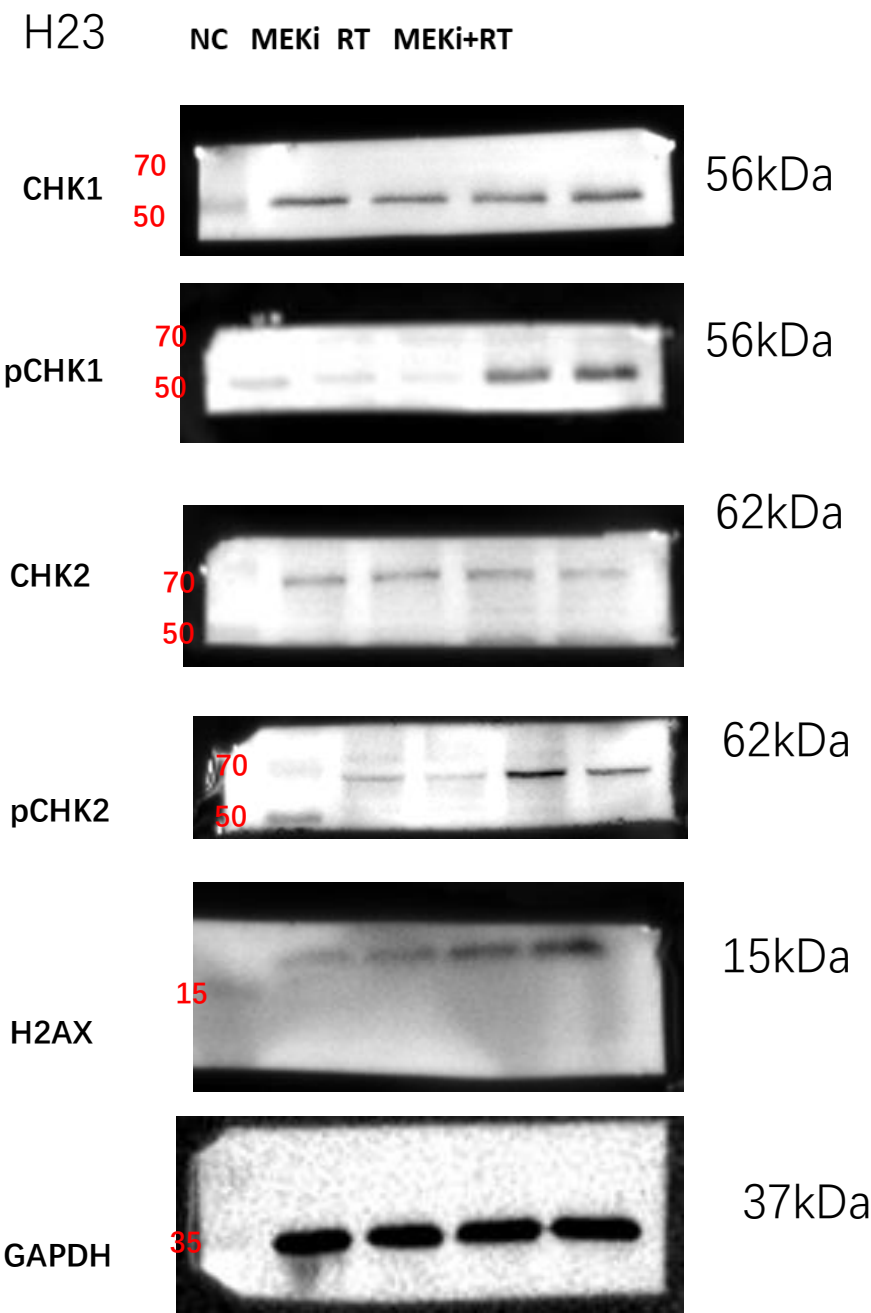

Figure 5C

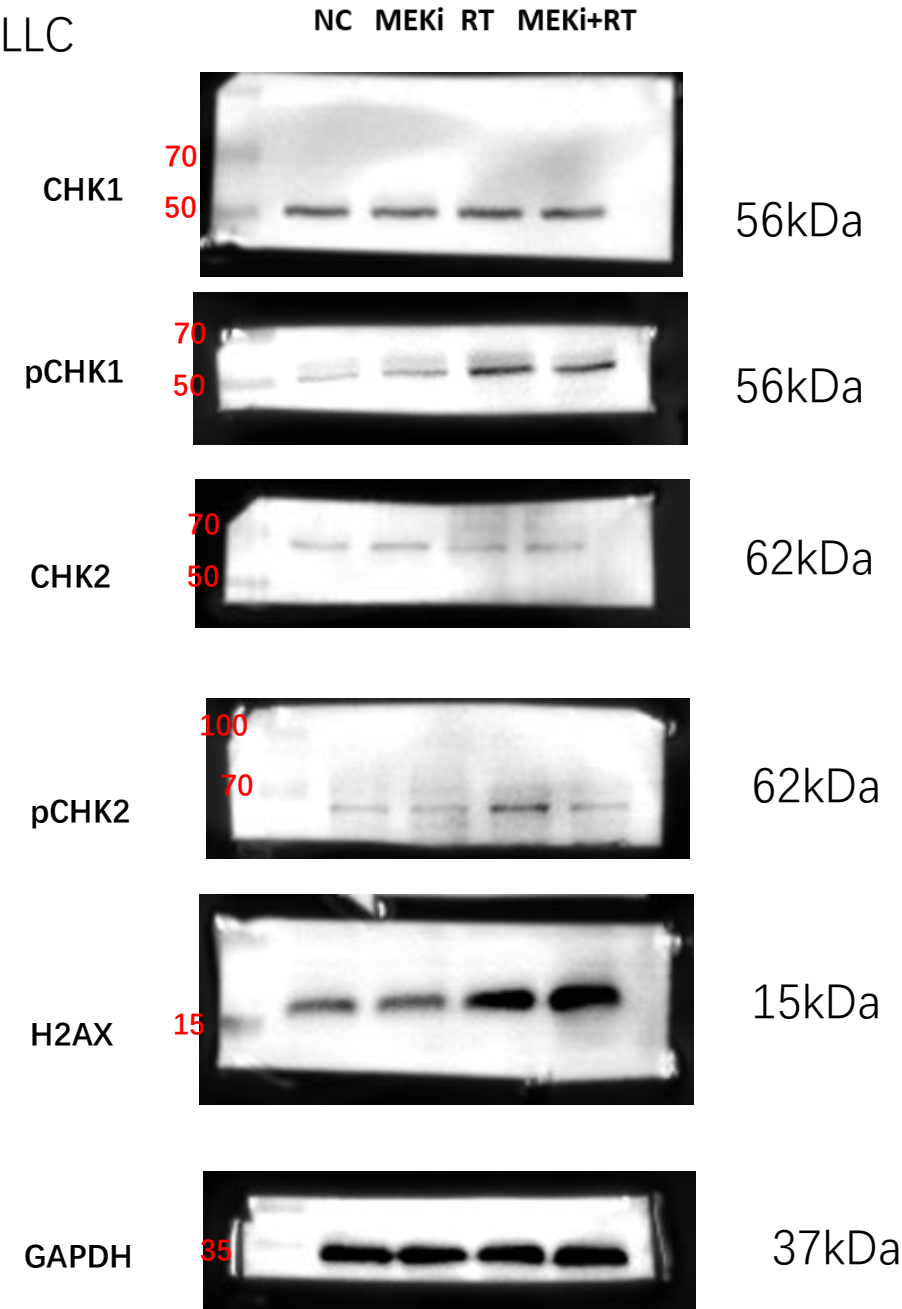

Supplement: Supplementary file 1 [file DataSheet1.pdf]

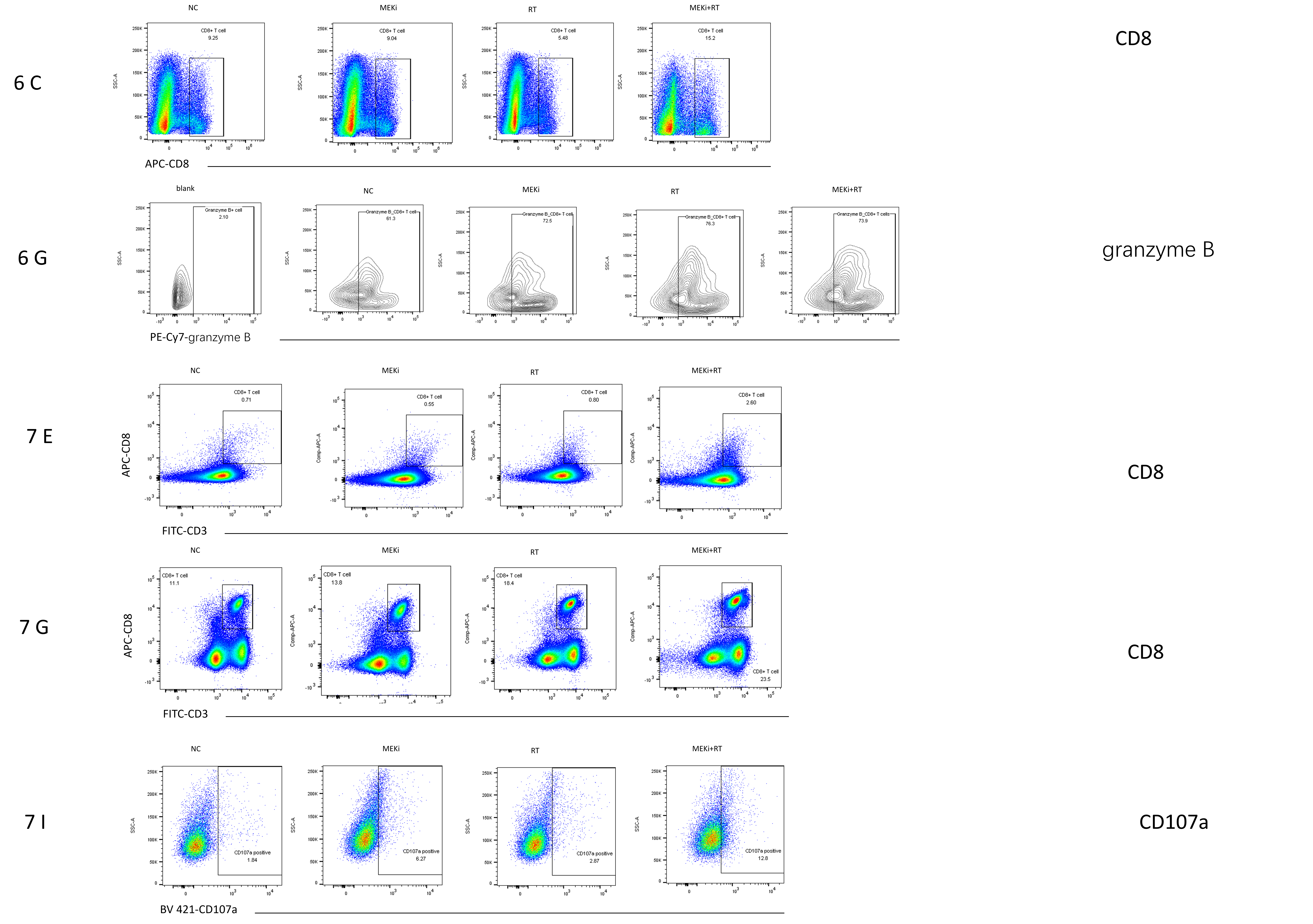

Supplement: Supplementary file 2 [file Image1.tif]
