# Supplementary material for: Impact of MEK inhibition on T-cell infiltration and function after radiotherapy in KRAS-mutant lung cancer
Source: Front Immunol. 2025 Nov 24;16:1663502. doi: 10.3389/fimmu.2025.1663502 (PMC12682748; doi:10.3389/fimmu.2025.1663502)
Supplement: Supplementary file 3 [file Table1.docx]

Supplementary Material

## Supplementary Figures


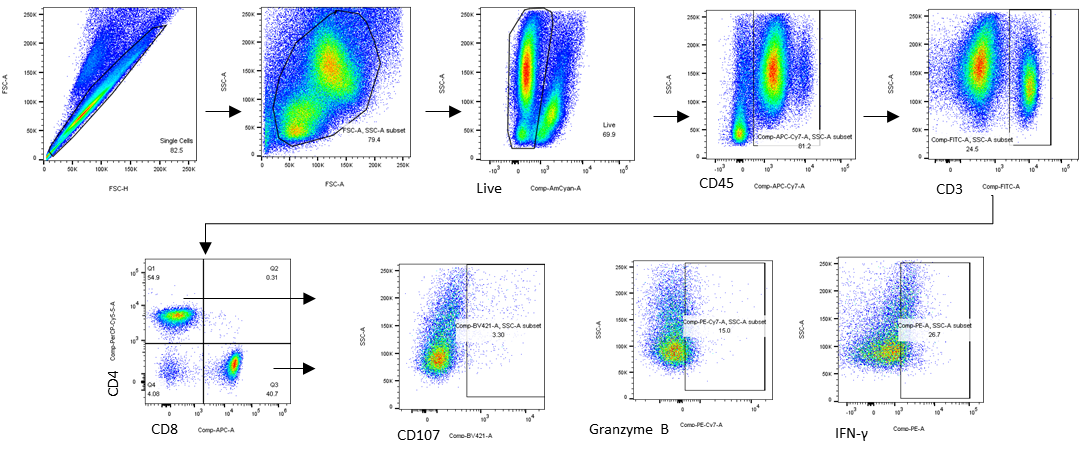


**Supplementary Figure 1.** **Demonstration of the gating strategy for T-cell analysis in spleen tissue**

**
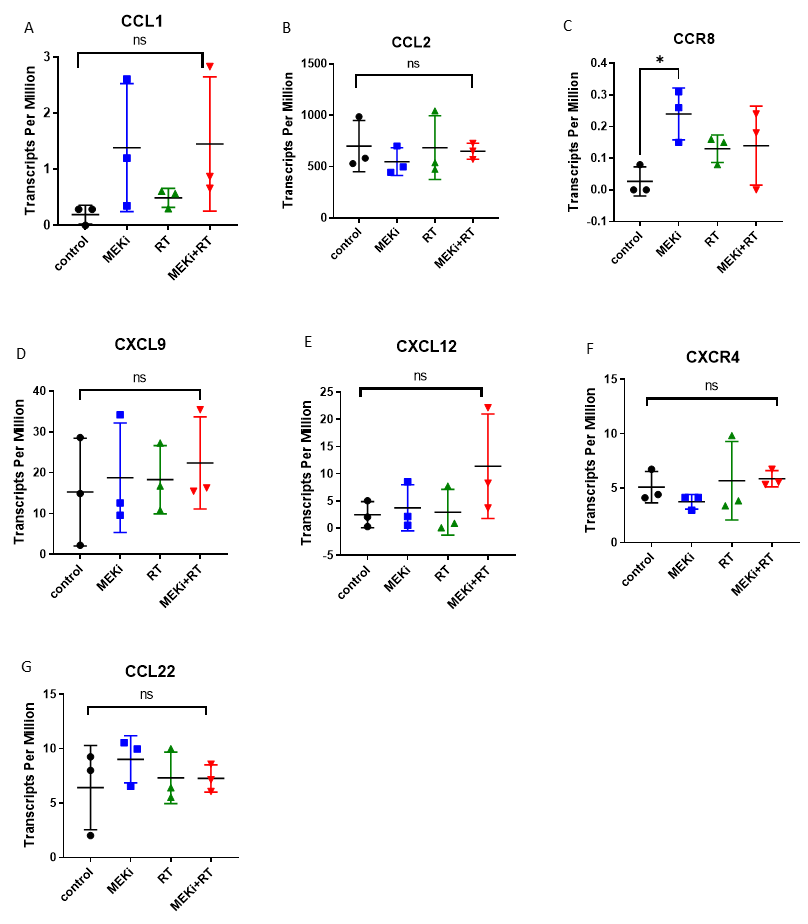
**

**Supplementary Figure 2. Analysis of sequencing data for the expression of chemokines and receptors**

**(**A-G) A comparison of the expression of CCL1, CCL2, CCR8, CXCL9, CXCL12, CXCR4, and CCL22

CCL: C-C motif chemokine ligand; CCR: C-C motif chemokine receptor; CXCL: C-X-C motif chemokine ligand; CXCR: C-X-C motif chemokine receptor

**
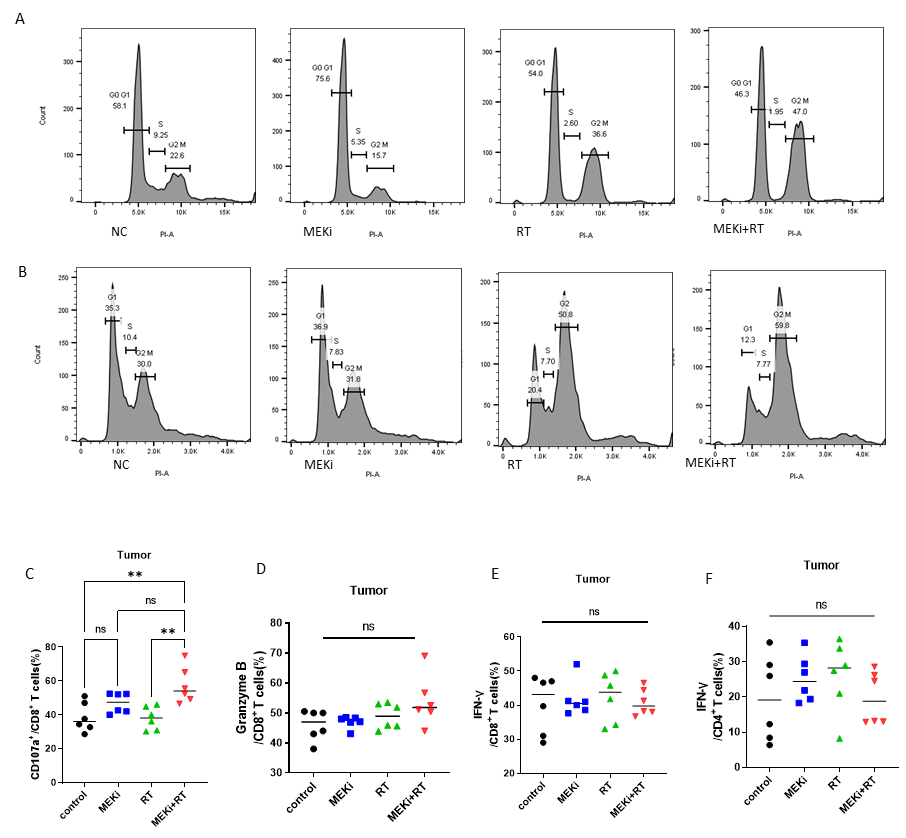
**

**Supplementary Figure 3.**

(A-B) Cell cycle analysis of A549 and H23 cells post-treatment. (C-F) Flow cytometry analysis of CD107a+, granzyme B, and IFN-γ+ CD8+ T lymphocytes and IFN-γ+ CD4+ T lymphocytes in tumor-infiltrating immune cells.

IFN: interferon
